# *In vivo* contrast free chronic myocardial infarction characterization using diffusion-weighted cardiovascular magnetic resonance

**DOI:** 10.1186/s12968-014-0068-y

**Published:** 2014-09-17

**Authors:** Christopher Nguyen, Zhaoyang Fan, Yibin Xie, James Dawkins, Eleni Tseliou, Xiaoming Bi, Behzad Sharif, Rohan Dharmakumar, Eduardo Marbán, Debiao Li

**Affiliations:** Biomedical Imaging Research Institute, Cedars-Sinai Medical Center, 116 N. Robertson Blvd Suite 800, Los Angeles, CA 90048 USA; Department of Bioengineering, University of California Los Angeles, 420 Westwood Plaza, Engineering V Room 5121, PO Box 951600, Los Angeles, CA 90095 USA; Heart Institute, Cedars-Sinai Medical Center, 127 S. San Vincente Blvd. Advanced Health Sciences Pavilion A3600, Los Angeles, CA 90048 USA; MR Research and Development, Siemens Healthcare, 116 N. Robertson Blvd Suite 800, Los Angeles, 90048 CA USA

## Abstract

**Background:**

Despite the established role of late gadolinium enhancement (LGE) cardiovascular magnetic resonance (CMR) in characterizing chronic myocardial infarction (MI), a significant portion of chronic MI patients are contraindicative for the use of contrast agents. One promising alternative contrast free technique is diffusion weighted CMR (dwCMR), which has been shown *ex vivo* to be sensitive to myocardial fibrosis. We used a recently developed *in vivo* dwCMR in chronic MI pigs to compare apparent diffusion coefficient (ADC) maps with LGE imaging for infarct characterization.

**Methods:**

In eleven mini pigs, chronic MI was induced by complete occlusion of the left anterior descending artery for 150 minutes. LGE, cine, and dwCMR imaging was performed 8 weeks post MI. ADC maps were derived from three orthogonal diffusion directions (b = 400 s/mm^2^) and one non-diffusion weighted image. Two semi-automatic infarct classification methods, threshold and full width half max (FWHM), were performed in both LGE and ADC maps. Regional wall motion (RWM) analysis was performed and compared to ADC maps to determine if any observed ADC change was significantly influenced by bulk motion.

**Results:**

ADC of chronic MI territories was significantly increased (threshold: 2.4 ± 0.3 μm^2^/ms, FWHM: 2.4 ± 0.2 μm^2^/ms) compared to remote myocardium (1.4 ± 0.3 μm^2^/ms). RWM was significantly reduced (threshold: 1.0 ± 0.4 mm, FWHM: 0.9 ± 0.4 mm) in infarcted regions delineated by ADC compared to remote myocardium (8.3 ± 0.1 mm). ADC-derived infarct volume and location had excellent agreement with LGE. Both LGE and ADC were in complete agreement when identifying transmural infarcts. Additionally, ADC was able to detect LGE-delineated infarcted segments with high sensitivity, specificity, PPV, and NPV. (threshold: 0.88, 0.93, 0.87, and 0.94, FWHM: 0.98, 0.97, 0.93, and 0.99, respectively).

**Conclusions:**

*In vivo* diffusion weighted CMR has potential as a contrast free alternative for LGE in characterizing chronic MI.

## Background

Measuring the size, location, and extent of myocardial infarction (MI) with cardiovascular magnetic resonance (CMR) has prognostic significance for the evaluation of left ventricular (LV) post-infarction remodeling [1,2]. Currently, the clinical gold standard to detect and characterize MI with CMR is late gadolinium enhanced (LGE) imaging, which requires the injection of an exogenous contrast agent. After a set delayed amount of time, gadolinium remains in infarcted tissue and shortens its T1 relaxation resulting in a large signal increase in T1-weighted images [3]. Despite its high clinical value, LGE is contraindicative for patients with severe renal disease, which constitutes a significant portion of MI patients [4–6]. CMR with endogenous contrasts such as T1ρ [7], creatine chemical exchange (CrEST) [8], native T1 [9–11], and water molecular diffusion [12,13], have recently demonstrated the potential to detect and characterize MI with strong correlation to LGE and histology.

Recent dwCMR techniques have addressed inherent challenges related to bulk motion artifacts with stimulated echo acquisition mode (STEAM) encoding [14,15], diffusion gradient moment nulling of spin echo encoding [16,17], ultrahigh gradient strength diffusion spin echo encoding [18], or continuous acquisition with temporal maximum projection filtered by principal component analysis [19]. Validation of the effectiveness of these techniques has largely been dependent on *in vivo* negative control comparisons, mechanical motion phantom experiments, and qualitative myofibril orientation comparisons. Although concordant results from these comparisons yield encouraging confidence in dwCMR techniques, there still exists some doubt in the performance of these techniques in detecting diseased tissue microstructure in the presence of complex cardiac bulk motion. Specifically, dwCMR could possibly still yield residual “bulk-motion weighting”, which could consequently confound any underlying tissue microstructure changes detected by dwCMR.

Previous *ex vivo* dwCMR studies in chronic MI porcine models have shown an increase in apparent diffusion coefficient (ADC) of infarcted regions and attributed this increase to a change in underlying tissue microstructure [12,13]. The origin of this tissue microstructure disarray was attributed to the presence of interstitial and replacement (infarct) myocardial fibrosis delineated by histology [13]. Furthermore, because the imaging studies were carried out *ex vivo* eight weeks post MI, the increase in ADC cannot be ascribed to edema and/or bulk motion. However for *in vivo* measurements, this significant increase in ADC due to the presence of myocardial fibrosis could either be diminished or completely absent if significant bulk-motion weighting is present. Consequently, the ability of detecting and characterizing infarction may be compromised.

The aim of this study was to determine if a recently developed *in vivo* dwCMR technique [16] could yield concordant infarct characterization with LGE in a chronic MI porcine model. This novel technique uses a second order motion compensation diffusion spin echo encoding with clinically available gradient strengths (~40 mT/m), which is distinctly different than conventional *in vivo* dwCMR STEAM methods that do not need motion compensation but instead rely on the periodicity between neighboring heartbeats. Additionally, regional wall motion was measured to verify that residual bulk-motion weighting did not significantly influence the ADC-based infarct characterization.

## Methods

### Animal preparation

Myocardial infarction was created in eleven Yucatan minipigs with the same preparation protocol as the control subjects of a previous study that validated LGE for *in vivo* infarct characterization [20]. Surgical cut-down to the left carotid artery and left jugular vein was performed. Anticoagulants with intravenous heparin were given to prevent blood clots forming on the catheters. Continuous administration of 2% lidocaine at 2 mg/kg and a single intravenous dose of amiodarone were administered. Prophylactic antibiotics were also administered intravenously. Coronary X-ray angiography was performed to visualize the coronary arteries and identify the site for coronary occlusion. A balloon dilation catheter (TREK®, Abbott Vascular) was inserted into the left anterior descending (LAD) coronary artery just below the first diagonal and inflated to achieve 100% occlusion of coronary blood flow for 150 minutes followed by reperfusion. Finally, the animal was taken to the post-op recovery room. All imaging was performed eight weeks post infarction.

### CMR protocol

To assess bulk motion and establish a reference percent infarct, cine bSSFP (TR/TE = 3.4/1.6, α = 50°, 35 cardiac phases, 1.4 × 1.4 × 6 mm^3^) and LGE using phase sensitive inversion recovery FLASH (TR/TE/TI = 326/1.47/300 ms, α = 20°, 1.3 × 1.3 × 6 mm^3^) imaging were performed on a 3 T system (MAGNETOM Verio, Siemens Healthcare, Erlangen, Germany) with a 12-channel phase array coil. All imaging was performed in the short axis plane at three slice locations (base, mid, and apex) of the LV. The cine imaging was performed prior to dwCMR using a single breath hold controlled by a ventilator for each slice. LGE imaging was completed 15 minutes after a dose (0.2 mmol/kg, gadoverstamide, Optimark, Mallinckrodt Inc, St. Louis, MO) of gadolinium was injected following dwCMR.

**Figure 1:**
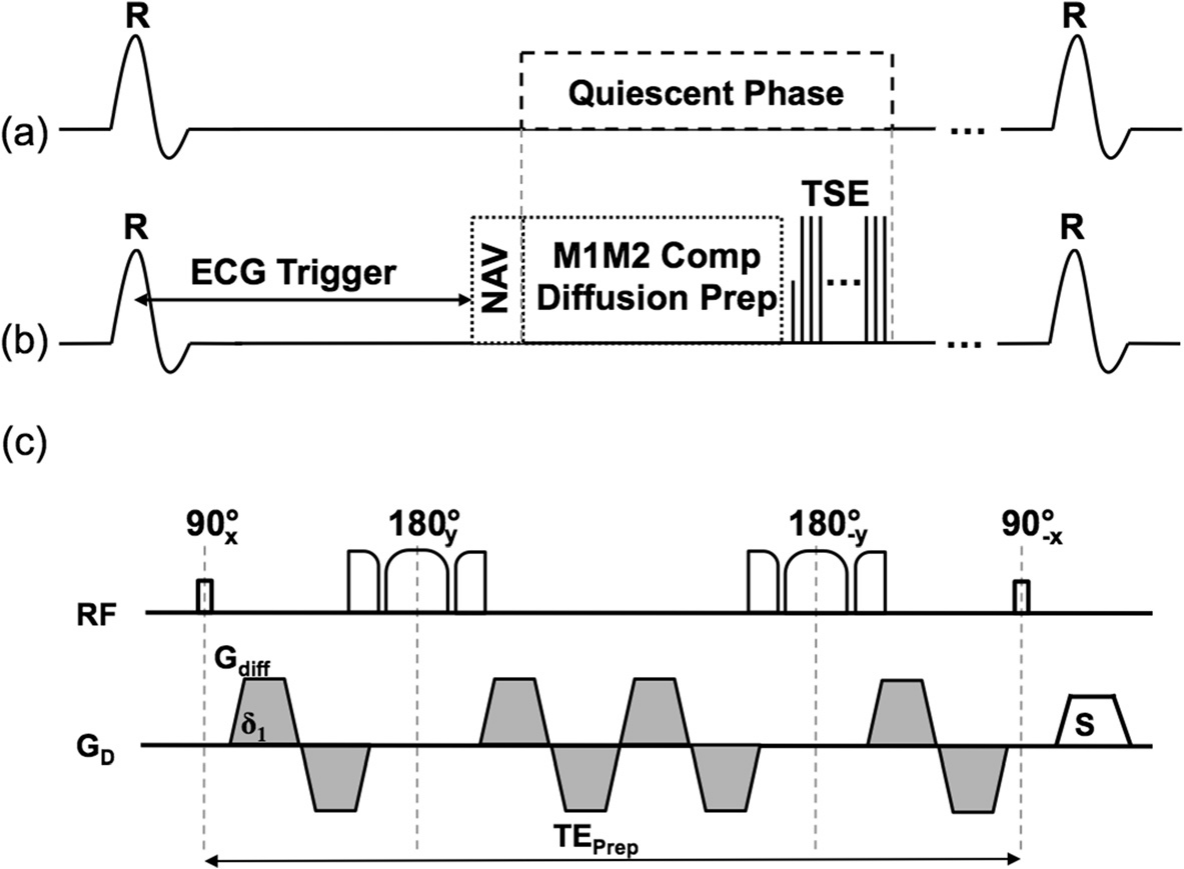
**Pulse sequence diagram of the navigator gated free breathing cardiac M1M2 compensated diffusion-prepared TSE technique.** The diffusion-prepared module and the segmented TSE readout **(b)** were placed in the quiescent period identified by cine **(a)**. The diffusion-prepared module **(c)** utilized non-selective BIR-4 adiabatic refocusing pulses to address expected 3 T B1-inhomogeneity and a second order gradient moment nulling scheme (quadra-bipolar).

A dwCMR sequence based off a recently developed diffusion-prepared sequence that utilizes both first and second order gradient moment nulling to compensate for bulk motion (M1M2) was performed (Figure 1) [16]. The M1M2 diffusion preparation (TE_prep_ = 105 ms) was achieved using a quadra-bipolar gradient scheme and BIR-4 adiabatic refocusing pulses to address B_1_-inhomogeneity [21]. Three orthogonal diffusion-weighted directions were respectively acquired at a b-value of 400 s/mm^2^ with a maximum gradient strength of 43 mT/m. The M1M2 diffusion preparation preceded a segmented turbo-spin echo (TSE) readout (TR/TE = 6RR/8.4 ms, α = 180°, echo spacing = 5.3 ms, 6 shots, 2.1 × 2.1 × 6 mm^3^) with matching slice thickness, number of slices, and FOV as cine and LGE imaging. Additionally, navigator-gating and ECG-triggering was performed. The diffusion preparation was applied at the beginning of the most quiescent period identified by cine imaging. In the case that TE_prep_ was greater than the quiescent period, then the number of TSE shots was doubled to avoid TSE-related cardiac motion artifacts. Scan time for each slice was approximately 3 minutes with about 50% navigator efficiency.

### Image analysis

ADC maps were calculated for each of the three diffusion directions (ADC_x_, ADC_y_, ADC_z_) using a 2-point fit to solve a monoexponential diffusion decay in Matlab (Mathworks, Natick, MA). A final trace apparent diffusion coefficient (ADC) map was calculated (ADC = [ADC_x_ + ADC_y_ + ADC_z_]/3). The mean ADC was calculated for each delineated infarct volume and a remote region defined as the lateral wall of the most basal short axis slice, which is furthest from the expected infarct that originates from the anteroseptal apex.

To assess bulk motion, the LV endocardium and epicardium were manually segmented for each cardiac phase image and for each phase, 100 evenly spaced chords were virtually defined. Regional wall motion (RWM) was calculated for each slice using commercially available software (Circle Cardiovascular Imaging Inc., Calgary, Canada) that determined the displacement of the myocardium along the defined chords. Wall motion was averaged over the chords that contained the ADC-derived infarct volume and the previously defined remote region.

To calculate infarct volume, two standard semi-automated approaches were explored to estimate the infarct volume, which was reported as a percentage normalized to the LV volume of the three acquired short-axis slices [22]. The first approach applied a simple threshold to identify infarct-containing voxels that have a signal intensity six standard deviations above the mean signal of a manually selected remote region-of-interest (ROI) [2]. The remote ROI was chosen to be same previously described basal lateral region. The second approach identified infarct-containing voxels by applying a region-growing algorithm that observes the full-width half maximum (FWHM) criterion. The FWHM criterion defines a “seed region” from a manually placed seed voxel that includes surrounding voxels ≥ 50% than the signal intensity of the seed point. It then determines the maximum intensity of that seed region and the final infarct region includes voxels that have signal intensity ≥ 50% of that maximum. The FWHM approach has demonstrated to have lower inter-observer bias, superior accuracy of infarct volume, and more immunity to variable post contrast delay time than the threshold approach [23].

**Figure 2:**
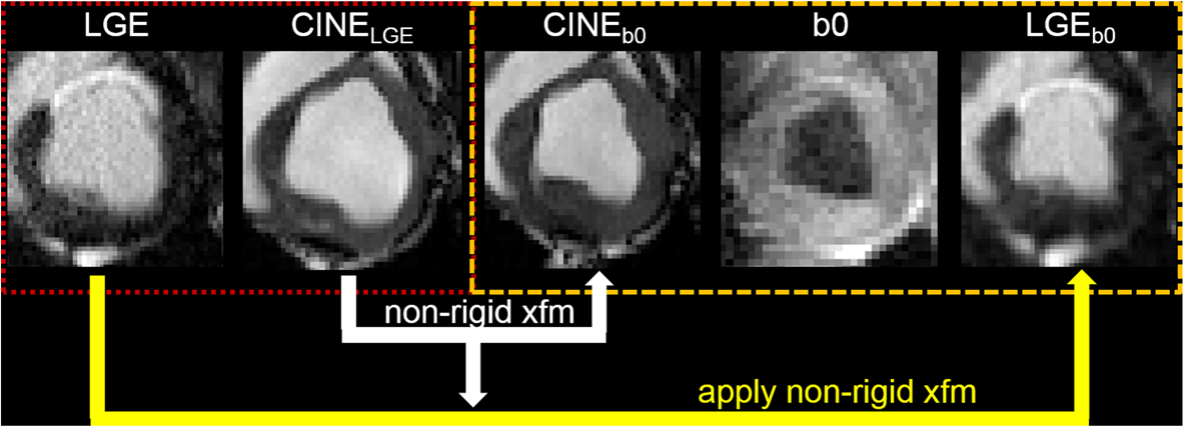
**Image co-registration scheme for infarct volume estimation.** Cine images were visually inspected to identify the two phases that matched the LGE and the least diffusion weighted image (b0), respectively. Non-rigid co-registration was performed on the Cine images to calculate the transform (xfm) from Cine_LGE_ (red box) to Cine_b0_ space (orange box). The calculated transform was then applied to the LGE image and the co-registered image (LGE_b0_) was used as an input for the two infarct volume estimation methods.

The two infarct volume estimation approaches were applied to LGE images and ADC maps. LGE images and ADC maps were co-registered to correct for cardiac phase mismatch before infarct volume was calculated (Figure 2). The co-registration was performed in a two-step process. First, the cine images were visually screened to identify the cardiac phase that closely resembled when the ADC map was acquired. This was repeated for the LGE image to find which cardiac phase it was acquired. Second, the two cardiac phases identified in the cine images were co-registered using non-rigid B-spline point-based registration [24]. The transform of that registration was applied to the LGE image. This ensured that co-registration did not affect the possible differences in infarct delineation if the LGE image was directly co-registered to the ADC map. The two infarct volume estimation approaches and the co-registration were implemented in Matlab.

For spatial location comparisons, each short axis slice was segmented into six standard American Heart Association (AHA) segments for the basal and mid short axis slices and four AHA segments for the apical slice. Segments positively identified to have infarct and/or akinetic wall motion were recorded in a binary manner. For ADC maps and LGE images, segments positive for infarct were defined as segments that contained any voxels that met the criteria defined by the threshold or FWHM methods. For RWM maps, akinetic segments were defined as segments with absolute wall displacement ≤ 3 mm [25]. Significance was tested for all segments as opposed to a per-segment basis and multiple comparisons where made between ADC and LGE. Sensitivity, specificity, positive predictive value (PPV), and negative predictive value (NPV) were calculated assuming LGE was the gold standard.

In addition, transmurality was qualitatively assessed for each found infarct by either threshold or FWHM criteria. Infarcts were characterized as being either completely transmural or not. ADC infarcts were visually matched to corresponding LGE infarcts if they were located within ±1 AHA segment of the ADC infarct. Subsequently, the binary infarct transmurality scoring was compared pairwise.

### Statistical analysis

All differences between means were statistically tested for significance using a Wilcoxon signed-rank test. To test for correspondence and agreement, Bland-Altman [26] and intraclass correlation (ICC) [27] analyses were performed. Although only telling of agreement, Pearson correlations (R^2^) were also reported. In the case of spatial location agreement that relied on a binary scoring, a Kappa test [28,29] was performed instead of Bland-Altman and intraclass correlation. Additionally to avoid potentially a Type 1 error a Bonferroni correction was performed manually to the significant difference testing of spatial location scores by lowering the significance limit to p < 0.0045. For all statistical tests, significance was denoted as p < 0.05 and the calculations were performed in Matlab. Finally, inter-observer reproducibility was tested in estimating infarct volume and location with two blinded reviewers defining the ROIs for remote myocardium for the threshold method and seed kernel for FWHM method. Intra-observer reproducibility was also tested in one of the blinded reviewers.

## Results

**Figure 3:**
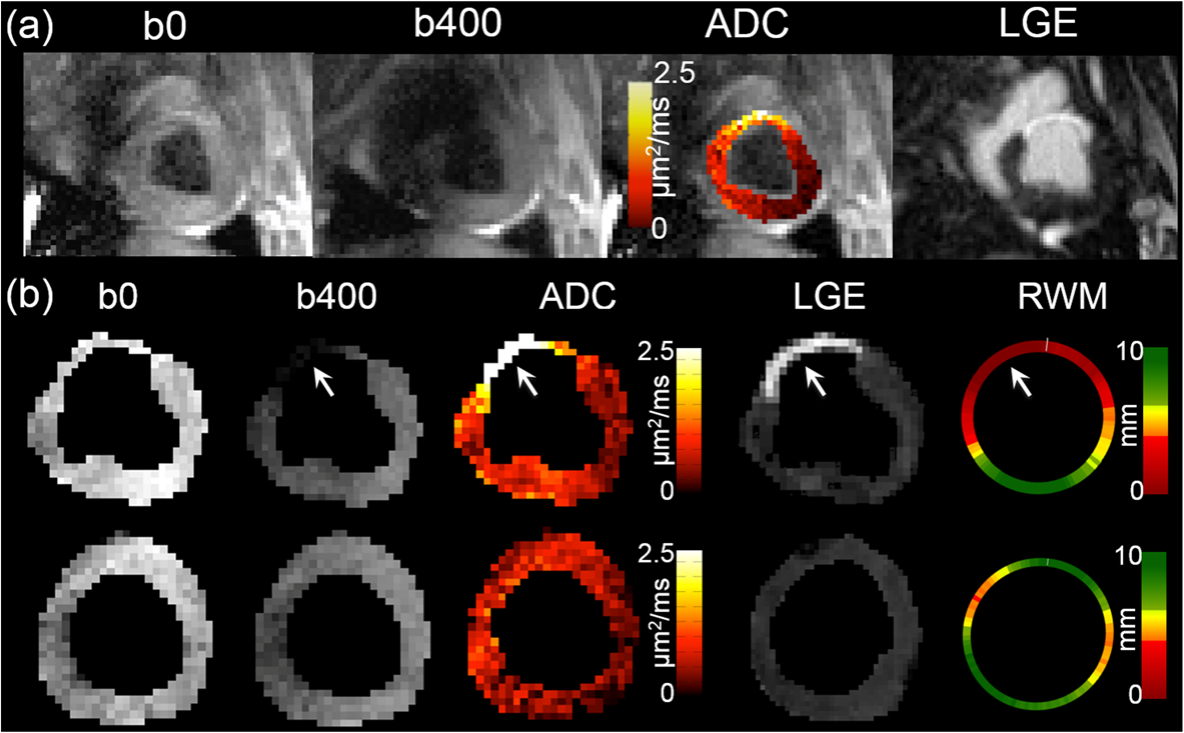
**Typical examples of DWI, ADC Map, LGE image, and RWM Map. (a)** Full field of view images of mean DWI (b0 and b400), ADC overlaid onto b0, and LGE shown to illustrate representative image quality. **(b)** Manual LV segmentation of two representative slices from another animal is shown containing infarct (top row) and no infarct (bottom row). Regions containing infarct defined by increased LGE signal had isointense T2 weighting (b0), hypointense diffusion weighting (b400), hyperintense ADC, and decreased RWM.

Using the threshold and FWHM criteria, infarct ADC was significantly increased (threshold: 2.4 ± 0.3 μm^2^/ms, FWHM: 2.4 ± 0.2 μm^2^/ms) compared to the ADC found in remote myocardium (1.4 ± 0.3 μm^2^/ms) (Table 1). RWM was significantly reduced (threshold: 1.0 ± 0.4 mm, FWHM: 0.9 ± 0.4 mm) in infarcted regions delineated by ADC compared to remote myocardium (8.3 ± 0.1 mm). Qualitatively, regions containing infarct defined by increased LGE signal had isointense T2 weighting (b0), hypointense diffusion weighting (b400), hyperintense ADC, and decreased RWM (Figure 3).

**Table 1:** **ADC and RWM of infarct and remote volumes**

	**Remote**	**Infarct**	
		**FWHM**	**Threshold**
ADC (μm^2^/ms)	1.4 ± 0.3	2.4** ± 0.2	2.4** ± 0.3
RWM (mm)	8.3 ± 0.1	0.9** ± 0.4	1.0** ± 0.4

**p < 0.001 Infarct vs Remote.

**Figure 4:**
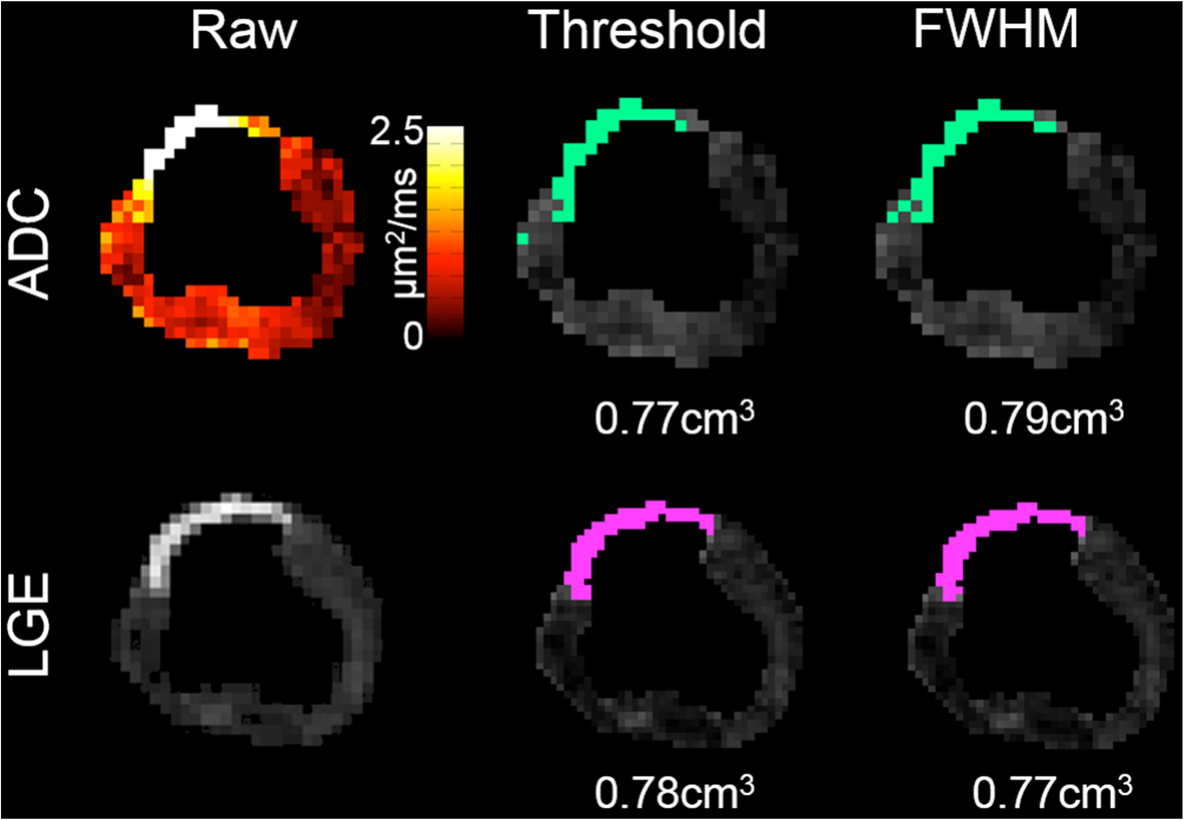
**Representative example of semi-automatic infarct volume estimation using threshold and FWHM criteria in LGE and ADC.** FWHM revealed better qualitative correspondence in infarct volume between LGE and ADC.

**Figure 5:**
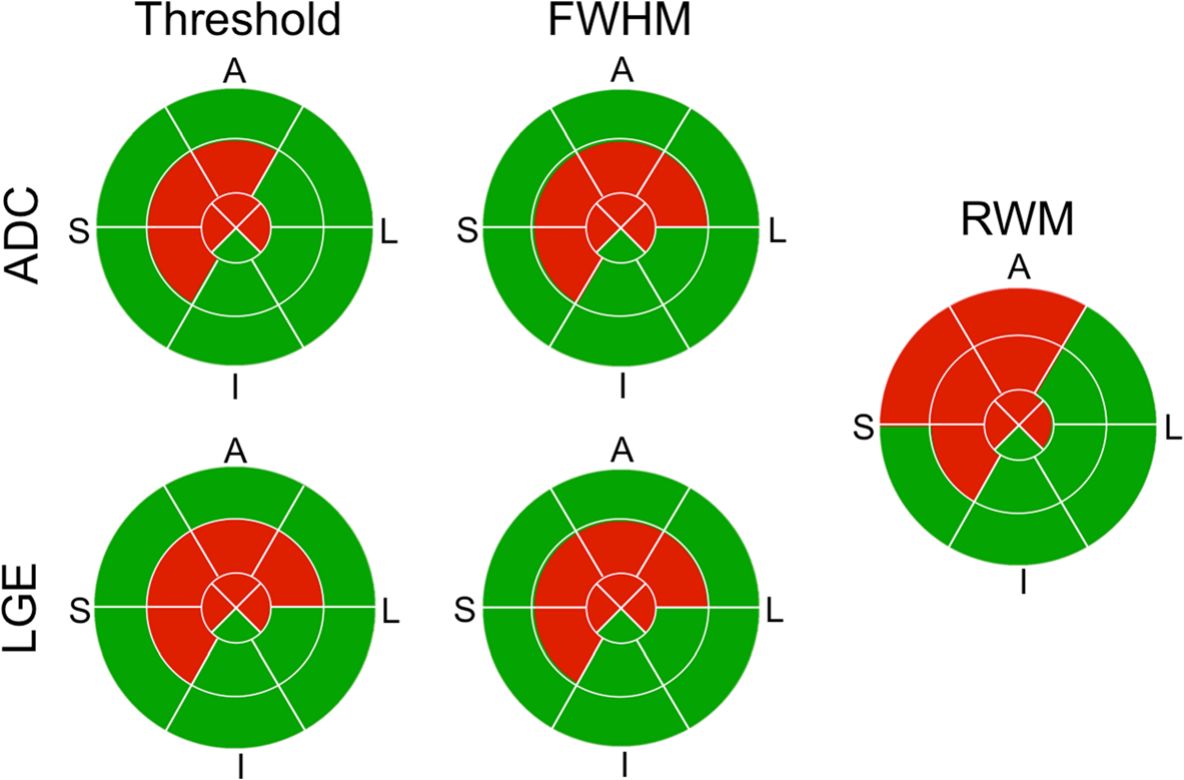
**Representative Bland-Altman and correlation plots of infarct volume comparison between LGE and ADC.** The Bland-Altman and correlation plots demonstrated excellent agreement between LGE and ADC using the two infarct volume estimations. Systemic biases were only qualitatively observed in the Bland-Altman plots. The Pearson correlations (R^2^) of the correlation plots were 0.97 and 0.99 for threshold and FWHM criteria, respectively.

ADC-derived infarct volume had excellent agreement with LGE-derived infarct volume (Figure 4 and Table 2). Bland-Altman plots (Figure 5) revealed minimal biases (<1.5%) for both threshold and FWHM criteria across both reviewers and repeated analyses. Additionally, the plots qualitatively presented systematic error with no indication of proportional or magnitude-related errors. ICC and R^2^ between ADC and LGE-derived infarct volumes were significantly high for both criteria (FWHM: all ICC > 0.95, threshold: all ICC > 0.93). This was reproducible between both blinded reviewers (ADC: ICC > 0.91, LGE: ICC > 0.94) and the repeated analysis of a single reviewer (ADC: ICC > 0.92, LGE: ICC > 0.94).

**Table 2:** **Infarct volume comparison of ADC and LGE**

		**Reviewer 1**		**Reviewer 1 Repeat**		**Reviewer 2**	
		**FWHM**	**Thresh**	**FWHM**	**Thresh**	**FWHM**	**Thresh**
LGE Infarct Volume (%)		11 ± 4	13 ± 4	11 ± 4	12 ± 5	10 ± 5	14 ± 4
ADC Infarct Volume (%)		11^++^ ± 4	12^++^ ± 4	11^++^ ± 3	13^++^ ± 4	9^++^ ± 4	13^++^ ± 4
ADC vs LGE	ICC	0.99**	0.98**	0.97**	0.95**	0.96**	0.93**
	R^2^	0.99	0.97	0.96	0.90	0.92	0.87
	bias (%)	0.2	0.2	0.2	−0.2	1.3	0.2
ADC R1 vs R2	ICC	--	--	--	--	0.91**	0.94**
	R^2^	--	--	--	--	0.85	0.90
	bias (%)	--	--	--	--	−1.9	0.8
LGE R1 vs R2	ICC	--	--	--	--	0.94**	0.97**
	R^2^	--	--	--	--	0.92	0.94
	bias (%)	--	--	--	--	−1.1	0.3
ADC R1 vs R1-re	ICC	--	--	0.96**	0.92**	--	--
	R^2^	--	--	0.92	0.86	--	--
	bias (%)	--	--	−0.6	1.2	--	--
LGE R1 vs R1-re	ICC	--	--	0.96**	0.94**	--	--
	R^2^	--	--	0.91	0.88	--	--
	bias (%)	--	--	0.3	−0.2	--	--

^++^not significant ADC vs LGE, **p < 0.01, thresh: threshold, R1: reviewer 1, R2: reviewer 2, R1-re: reviewer 1 repeat.

**Figure 6:**
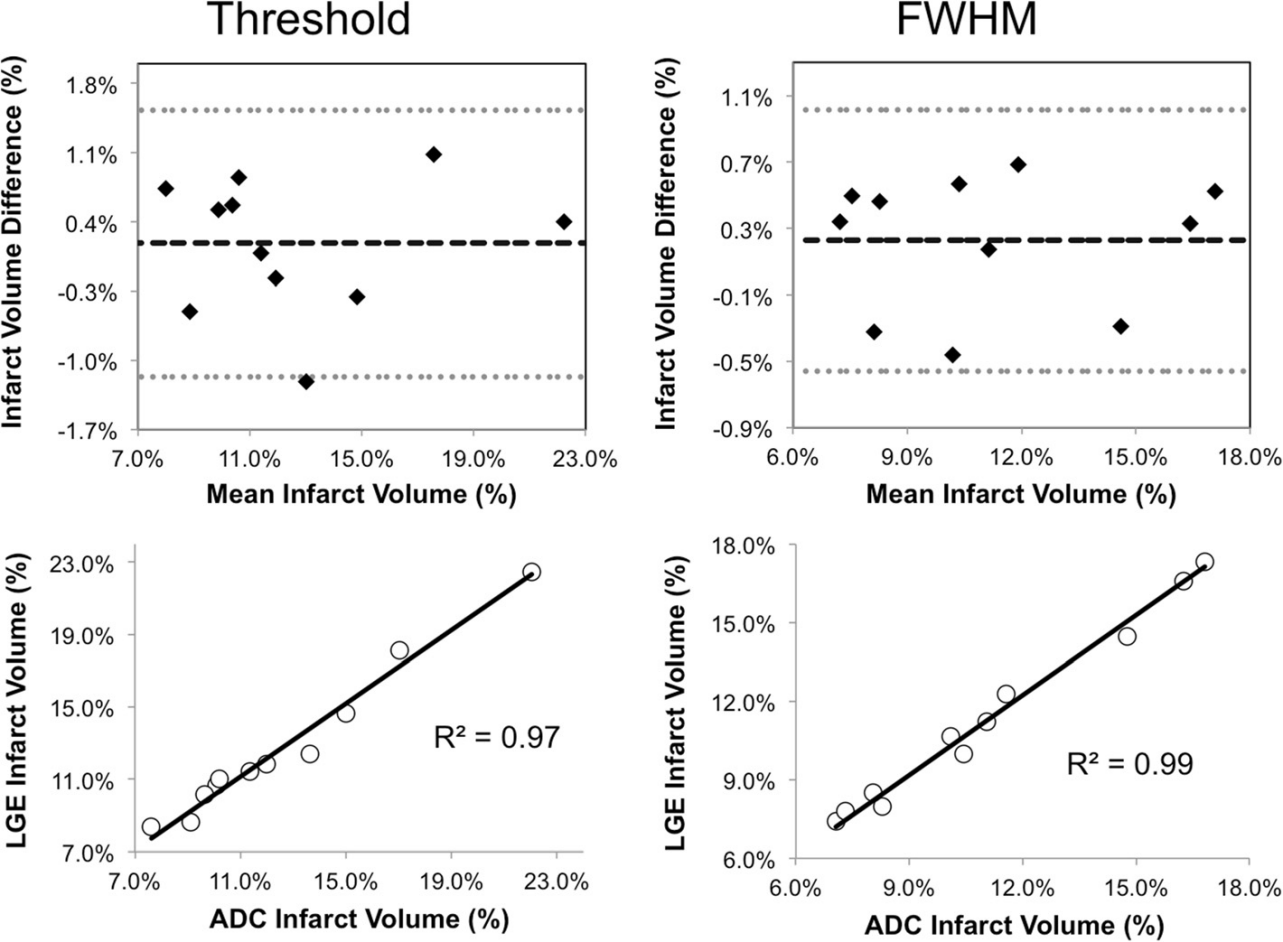
**Representative example of infarct location comparison of ADC, LGE, and RWM.** The binary scoring (red: positive, green: negative) of the 16 segment AHA wheels between ADC and LGE depicted substantial agreement using the FWHM (100% segments match) and threshold criteria (94% segments match). The region with abnormal wall motion (8 segments) was greater than the infarct region delineated by ADC (threshold: 6 segments, FWHM: 7 segments) and LGE (threshold: 7 segments, FWHM: 7 segments).

Infarct location comparisons of the AHA segmentation revealed substantial agreement (threshold: κ > 0.80, FWHM: all κ > 0.93, for all p < 0.01) between LGE and ADC for both reviewers (Figure 6 and Table 3). The percentage of segments in agreement was ≥ 90% and no significant difference was observed for both infarct volume criteria. ADC was able to detect LGE-delineated infarcts with high sensitivity, specificity, PPV, and NPV (Reviewer #1 threshold: 0.88, 0.93, 0.87, and 0.94, FWHM: 0.98, 0.97, 0.93, and 0.99; Reviewer #2 threshold: 0.89, 0.92, 0.85, and 0.95, FWHM: 0.96, 0.98, 0.96, and 0.98, respectively). The number of segments with abnormal RWM (n = 81) was significantly (p < 0.001) greater than the number of infarcted segments for ADC (Reviewer #1: threshold: n = 60, FWHM: n = 55, Reviewer #2: threshold: n = 62, FWHM: n = 47) and LGE (Reviewer #1: threshold: n = 59, FWHM: n = 52; Reviewer #2: threshold: n = 59, FWHM: n = 47). Segments with abnormal RWM encapsulate the locations identified for infarct by both ADC and LGE for all infarct volume criteria. These results were highly reproducible with no difference in infarct location between repeated analysis and substantial agreement (ADC: threshold: κ = 0.90, FWHM: κ = 0.94; LGE: threshold: κ = 0.95, FWHM: κ = 0.97) between the two reviewers.

**Table 3:** **Infarct location comparison of ADC and LGE**

		**Reviewer #1**				**Reviewer #2**			
		**LGE**				**LGE**			
		**FWHM**		**Threshold**		**FWHM**		**Threshold**	
		+	-	+	-	+	-	+	-
ADC	+	51	1	52	7	45	2	53	6
	-	4	120	8	109	2	127	9	108
# segs in agreement		171 (97%)		161 (92%)		172 (98%)		161 (91%)	
Cohen’s Kappa (κ)		0.93**		0.81**		0.94**		0.81**	
Wilcoxon test (*p*)		NS		NS		NS		NS	
# LGE infarct segs		55 (31%)		60 (34%)		47 (27%)		62 (35%)	
# ADC infarct segs		52 (30%)		59 (34%)		47 (27%)		59 (34%)	
Sensitivity^++^		0.98		0.88		0.96		0.89	
Specificity^++^		0.97		0.93		0.98		0.92	
PPV^++^		0.93		0.87		0.96		0.85	
NPV^++^		0.99		0.94		0.98		0.95	

**p < 0.0001, NS: not significant, ^++^LGE was gold standard, segs: segments.

Transmural infarcts were observed in all eleven animals. Both LGE and ADC were in complete agreement under threshold and FWHM criteria in identifying the infarcts as having a fully transmural characteristic.

## Discussion

Using a recently developed *in vivo* dwCMR technique, we have demonstrated in a large animal model of chronic MI that ADC was significantly increased in infarcted regions compared to remote myocardium. A chronic MI porcine model was chosen because previous *ex vivo* dwCMR studies established that the ADC increase found in chronic infarcts was due to the underlying tissue microstructural presence of myocardial fibrosis. Additionally, the presence of edema would be absent leaving only bulk motion as the major confounding source of an observed *in vivo* ADC increase. Infarct quantification utilized two common semi-automatic approaches, threshold and FWHM, to avoid potential inter-observer bias and provide a more quantitative infarct characterization. RWM analysis was performed and compared to ADC maps to determine if any observed ADC change was significantly influenced by bulk motion.

ADC estimation of infarct volume and location demonstrated substantial agreement and correspondence with LGE. These findings were highly reproducible between two blinded reviewers and repeated analysis of a single reviewer. In yielding the best agreement between ADC and LGE, FWHM outperformed the threshold method and yielded better of inter- and intra-observer reproducibility. In the infarcted regions identified by LGE, ADC was significantly increased while RWM was significantly decreased when compared with remote myocardium. In the regions with elevated ADC, the tissue’s bulk motion assessed by cine imaging was significantly reduced compared with remote regions. Consequently, if residual bulk-motion weighting were to exist and dominated the overall change in ADC, then the expected ADC would have instead been decreased relative to remote regions as opposed to the observed increase. Furthermore, high intraclass correlation and agreement in estimating infarct volume when compared to LGE suggests that bulk motion has little effect on the observed increase in ADC. A more rigorous follow-up experiment that includes applying dwCMR immediately before and after the myocardium is arrested will be needed to conclusively demonstrate that an *in vivo* dwCMR technique is completely free of bulk motion. Such an experiment at the time of our study was logistically infeasible. Additionally, T2 weighting visualized by the least diffusion-weighted measurement (b0) depicted isointensity between the infarct and remote regions. Therefore, the results strongly suggest that the observed increase in ADC found in infarct regions was a reflection of underlying tissue microstructure change as opposed to the presence of bulk motion or edema [30].

The significant increase in ADC (~70%) found in the study is consistent with the significant increases found in previous *ex vivo* studies that used similar large animal models (50-80%) [12,13]. However, the absolute infarct (2.3 μm^2^/ms) and remote (1.4 μm^2^/ms) myocardial ADC values are larger than what was presented *ex vivo* (Pop, *et al.*: 1.1 μm^2^/ms infarct vs 0.62 μm^2^/ms remote, Wu, *et al.*: 1.0 μm^2^/ms infarct vs 0.67 μm^2^/ms remote). This disparity could be attributed to the use of formalin, dehydration, and differences in temperature, which are known to perturb absolute ADC values in *ex vivo* conditions [31]. In a recent *in vivo* chronic MI patient study [32], infarct ADC was also significantly increased, but did not exhibit the same large relative increase (~10%) as the work presented. This is most likely due to the variety in presentation of chronic infarction in the patients recruited, in which some did not receive any percutaneous intervention while others did. This is in comparison to the 150 min LAD occlusion and reperfusion of the animal model used in this study. A follow-up study using the diffusion-prepared TSE technique in a similar patient demographic needs to be conducted to confirm that the large relative increase in ADC found in this study is present in chronic MI patients as well.

Despite the potential for ADC to quantify the degree of fibrosis shown by Pop, *et al.*, the *in vivo* quantification of ADC in this study cannot precisely predict the severity of fibrosis because the degree of bulk motion weighting was not fully quantified. Cine imaging used in this study can only qualitatively yield the radial displacement of the myocardium as opposed to more sophisticated myocardial strain or phase contrast tissue mapping [33], which can quantitatively measure bulk motion entirely. Therefore, the observed elevated ADC can currently only detect the presence of fibrosis similar to LGE imaging. A future study using myocardial strain or phase contrast tissue mapping to discern the exact amount of bulk motion weighting affecting the proposed *in vivo* dwCMR technique should be conducted in combination with the aforementioned arrested myocardium experiment.

Limitations of the diffusion-prepared TSE technique used in this study include potentially long scan times and low spatial coverage. Although the TSE readout generally yields less image artifacts than balanced steady-state free precession at 3 T, it unfortunately has longer echo spacing leading to an overall two to three times increase in scan time. Therefore to keep the imaging time to a manageable 10 minutes, the spatial coverage was reduced to three slices. Consequently, the infarct volume estimated in this study was not calculated from the whole LV potentially yielding a deficient estimate of the true infarct volume. However, the focus of this study was on the comparison of ADC with LGE and despite the limited spatial coverage, the two yielded comparable estimates in infarct volume. Furthermore since the diffusion-preparation approach is inherently multi-shot, both scan time and spatial coverage limitations could be overcome with a 3D whole-heart radial acquisition with parallel imaging and iterative reconstruction [34]. Potentially, this technique could markedly reduce the scan time while providing whole-heart coverage.

Another limitation of this study was that the animal model used only yielded transmural infarcts at the LAD territory. Consequently, the ability to detect subendocardial infarction with dwCMR was not tested, which is often of clinical importance in viability imaging of chronic MI. Non-transmural infarcts are generally much smaller in size, which could be more difficult to detect with the lower spatial resolution of dwCMR. Additionally, infarction was only induced in the LAD territory, which limits the study in testing the performance of the new technique to locate infarction in other arterial territories. Further studies will be needed to specifically investigate the ability of dwCMR ability to detect non-transmural infarction at different arterial territories.

## Conclusions

The concordance between ADC and LGE combined with presence of abnormal RWM strongly suggests that the observed increased ADC reflects changes in underlying myocardial tissue microstructure. Consequently, *in vivo* diffusion weighted CMR has the potential to serve as a contrast free alternative for LGE in characterizing chronic MI.
